# 1,4-Bis(hex­yloxy)benzene

**DOI:** 10.1107/S1600536813029024

**Published:** 2013-10-31

**Authors:** Hua Cheng

**Affiliations:** a College of Chemical Engineering and Food Science, Hubei University of Arts and Science, Xiangyang 441053, People’s Republic of China

## Abstract

The asymmetric unit of the title compound, C_18_H_30_O_2_, contains one half-mol­ecule situated on an inversion center. The alkyl chain adopts a fully extended all-*trans* conformation. The C atoms of the alkyl chain are almost coplanar, with a maximum deviation of 0.042 (6) Å from the mean plane,which is inclined to the central benzene ring by 6.80 (9)°. The crystal packing exhibits no short inter­molecular contacts.

## Related literature

For the synthesis and applications of the title compound, see: Ramesh & Thomas (2010 ▶); Mayor & Didschies (2003 ▶); Choi *et al.* (2006 ▶). For the crystal structures of related compounds, see: Li *et al.* (2008 ▶); Thevenet *et al.* (2010 ▶).
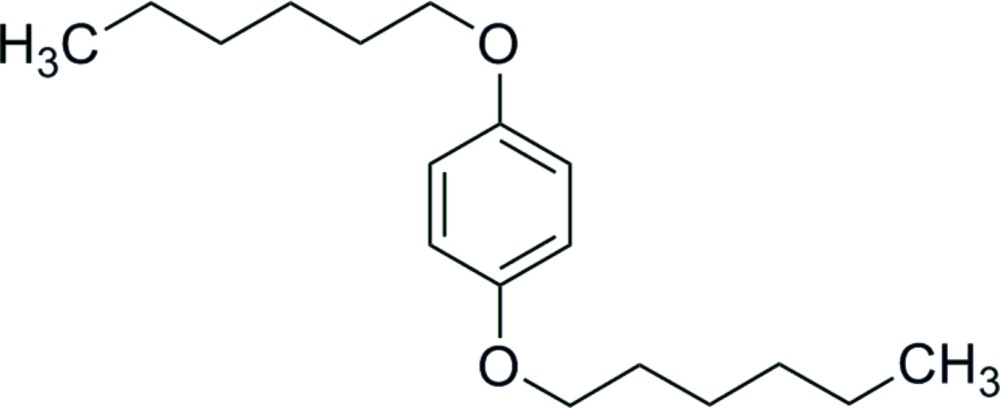


## Experimental

### 

#### Crystal data

C_18_H_30_O_2_
*M* *_r_* = 278.42
Monoclinic, 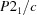

*a* = 18.853 (12) Å
*b* = 7.512 (5) Å
*c* = 6.364 (4) Å
β = 95.674 (10)°
*V* = 896.9 (11) Å^3^
*Z* = 2
Mo *K*α radiation
μ = 0.07 mm^−1^
*T* = 298 K
0.06 × 0.05 × 0.04 mm

#### Data collection

Bruker APEXII CCD diffractometer
Absorption correction: multi-scan (*SADABS*; Bruker, 2001 ▶) *T*_min_ = 0.996, *T*_max_ = 0.997
6024 measured reflections
1552 independent reflections
760 reflections with *I* > 2σ(*I*)
*R*_int_ = 0.099

#### Refinement

*R*[*F* ^2^ > 2σ(*F* ^2^)] = 0.067
*wR*(*F* ^2^) = 0.238
*S* = 0.99
1552 reflections
92 parameters
H-atom parameters constrained
Δρ_max_ = 0.17 e Å^−3^
Δρ_min_ = −0.15 e Å^−3^

### 

Data collection: *APEX2* (Bruker, 2001 ▶); cell refinement: *SAINT* (Bruker, 2001 ▶); data reduction: *SAINT*; program(s) used to solve structure: *SHELXS97* (Sheldrick, 2008 ▶); program(s) used to refine structure: *SHELXL97* (Sheldrick, 2008 ▶); molecular graphics: *SHELXTL* (Sheldrick, 2008 ▶); software used to prepare material for publication: *SHELXTL*.

## Supplementary Material

Crystal structure: contains datablock(s) I, global. DOI: 10.1107/S1600536813029024/cv5434sup1.cif

Structure factors: contains datablock(s) I. DOI: 10.1107/S1600536813029024/cv5434Isup2.hkl

Supplementary material file. DOI: 10.1107/S1600536813029024/cv5434Isup3.cml

Additional supplementary materials:  crystallographic information; 3D view; checkCIF report

## Figures and Tables

**Fig. 1.**
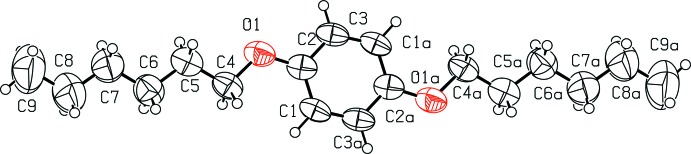
The title molecule with the atom-numbering scheme and 50% probability displacement ellipsoids [symmetry code: (a) -x, 1 -y, 2 -z].

## supplementary crystallographic information

### 1. Comment

The title compound is an important intermediate in the synthesis of conjugated polymers (Mayor & Didschies, 2003; Choi *et al.*, 2006) and supramolecular networks (Ramesh & Thomas, 2010). Herein we report its crystal structure.

In the molecule (Fig. 1), the alkyl chain adopts a fully extended all-*trans* conformation. The C-atoms of the alkyl chain are almost coplanar with the maximum deviation of 0.042 (6) Å from the mean plane, and this mean plane is inclined to the central benzene ring by 6.80 (9)°. The crystal packing exhibits no short intermolecular contacts.

### 2. Experimental

The title compound was synthesized according to the known method (Ramesh & Thomas, 2010). Crystals suitable for single-crystal X-ray diffraction were grown by slow evaporation of the solution in hexane-MeOH (5:1).

### 3. Refinement

All H atoms were positioned geometrically (C—H = 0.93–0.97 Å) and refined as riding, allowing for free rotation of the methyl groups. The constraint *U*_iso_(H) = 1.2 *U*_eq_(C) or 1.5 *U*_eq_(C) (methyl C) was applied.

### Crystal data

**Table:**

C_18_H_30_O_2_	*F*(000) = 308
*M**_r_* = 278.42	*D*_x_ = 1.031 Mg m^−^^3^
Monoclinic, *P*2_1_/*c*	Mo *K*α radiation, λ = 0.71073 Å
Hall symbol: -P 2ybc	Cell parameters from 885 reflections
*a* = 18.853 (12) Å	θ = 2.2–21.8°
*b* = 7.512 (5) Å	µ = 0.07 mm^−^^1^
*c* = 6.364 (4) Å	*T* = 298 K
β = 95.674 (10)°	Block, colourless
*V* = 896.9 (11) Å^3^	0.06 × 0.05 × 0.04 mm
*Z* = 2	

### Data collection

**Table:**

Bruker APEXII CCD diffractometer	1552 independent reflections
Radiation source: fine-focus sealed tube	760 reflections with *I* > 2σ(*I*)
Graphite monochromator	*R*_int_ = 0.099
φ and ω scans	θ_max_ = 25.0°, θ_min_ = 2.2°
Absorption correction: multi-scan (*SADABS*; Bruker, 2001)	*h* = −22→21
*T*_min_ = 0.996, *T*_max_ = 0.997	*k* = −8→8
6024 measured reflections	*l* = −7→7

### Refinement

**Table:**

Refinement on *F*^2^	Primary atom site location: structure-invariant direct methods
Least-squares matrix: full	Secondary atom site location: difference Fourier map
*R*[*F*^2^ > 2σ(*F*^2^)] = 0.067	Hydrogen site location: inferred from neighbouring sites
*wR*(*F*^2^) = 0.238	H-atom parameters constrained
*S* = 0.99	*w* = 1/[σ^2^(*F*_o_^2^) + (0.1237*P*)^2^] where *P* = (*F*_o_^2^ + 2*F*_c_^2^)/3
1552 reflections	(Δ/σ)_max_ < 0.001
92 parameters	Δρ_max_ = 0.17 e Å^−^^3^
0 restraints	Δρ_min_ = −0.15 e Å^−^^3^

### Special details

**Table:**

Geometry. All e.s.d.'s (except the e.s.d. in the dihedral angle between two l.s. planes) are estimated using the full covariance matrix. The cell e.s.d.'s are taken into account individually in the estimation of e.s.d.'s in distances, angles and torsion angles; correlations between e.s.d.'s in cell parameters are only used when they are defined by crystal symmetry. An approximate (isotropic) treatment of cell e.s.d.'s is used for estimating e.s.d.'s involving l.s. planes.
Refinement. Refinement of *F*^2^ against ALL reflections. The weighted *R*-factor *wR* and goodness of fit *S* are based on *F*^2^, conventional *R*-factors *R* are based on *F*, with *F* set to zero for negative *F*^2^. The threshold expression of *F*^2^ > σ(*F*^2^) is used only for calculating *R*-factors(gt) *etc*. and is not relevant to the choice of reflections for refinement. *R*-factors based on *F*^2^ are statistically about twice as large as those based on *F*, and *R*- factors based on ALL data will be even larger.

### Fractional atomic coordinates and isotropic or equivalent isotropic displacement parameters (Å^2^)

**Table:**

	*x*	*y*	*z*	*U*_iso_*/*U*_eq_	
C1	0.01445 (17)	0.4133 (3)	0.8176 (4)	0.0693 (9)	
H1	0.0241	0.3542	0.6952	0.083*	
C2	0.06718 (16)	0.5102 (3)	0.9318 (4)	0.0664 (8)	
C3	0.05200 (17)	0.5954 (4)	1.1147 (4)	0.0700 (8)	
H3	0.0876	0.6597	1.1928	0.084*	
C4	0.15191 (16)	0.4640 (4)	0.6777 (4)	0.0795 (9)	
H4A	0.1171	0.5055	0.5661	0.095*	
H4B	0.1514	0.3348	0.6782	0.095*	
C5	0.22423 (18)	0.5306 (5)	0.6427 (5)	0.0905 (10)	
H5A	0.2228	0.6595	0.6360	0.109*	
H5B	0.2571	0.4978	0.7634	0.109*	
C6	0.25233 (18)	0.4623 (5)	0.4483 (5)	0.0959 (11)	
H6A	0.2207	0.4996	0.3269	0.115*	
H6B	0.2520	0.3332	0.4520	0.115*	
C7	0.3266 (2)	0.5246 (5)	0.4201 (6)	0.1107 (13)	
H7A	0.3262	0.6535	0.4098	0.133*	
H7B	0.3576	0.4929	0.5452	0.133*	
C8	0.3570 (3)	0.4521 (8)	0.2347 (7)	0.1542 (19)	
H8A	0.3263	0.4849	0.1095	0.185*	
H8B	0.3569	0.3232	0.2443	0.185*	
C9	0.4300 (3)	0.5113 (10)	0.2076 (9)	0.202 (3)	
H9A	0.4313	0.6389	0.2018	0.303*	
H9B	0.4444	0.4631	0.0787	0.303*	
H9C	0.4619	0.4704	0.3245	0.303*	
O1	0.13529 (11)	0.5307 (3)	0.8769 (3)	0.0818 (8)	

### Atomic displacement parameters (Å^2^)

**Table:**

	*U*^11^	*U*^22^	*U*^33^	*U*^12^	*U*^13^	*U*^23^
C1	0.106 (2)	0.0549 (16)	0.0456 (15)	−0.0004 (15)	0.0022 (15)	−0.0034 (12)
C2	0.089 (2)	0.0570 (17)	0.0516 (15)	−0.0033 (13)	−0.0020 (13)	0.0104 (13)
C3	0.103 (2)	0.0531 (16)	0.0516 (15)	−0.0086 (14)	−0.0060 (13)	0.0021 (12)
C4	0.099 (2)	0.0787 (19)	0.0600 (18)	0.0063 (16)	0.0020 (14)	−0.0041 (15)
C5	0.105 (2)	0.085 (2)	0.081 (2)	0.0025 (18)	0.0044 (16)	−0.0080 (17)
C6	0.100 (3)	0.102 (3)	0.086 (2)	0.0084 (19)	0.0097 (17)	−0.0030 (19)
C7	0.108 (3)	0.116 (3)	0.108 (3)	0.002 (2)	0.013 (2)	−0.002 (2)
C8	0.140 (4)	0.200 (5)	0.127 (4)	0.002 (4)	0.041 (3)	−0.017 (4)
C9	0.140 (4)	0.272 (9)	0.205 (6)	−0.003 (4)	0.070 (4)	−0.004 (5)
O1	0.0997 (16)	0.0855 (15)	0.0591 (12)	−0.0089 (11)	0.0017 (10)	−0.0092 (10)

### Geometric parameters (Å, º)

**Table:**

C1—C3^i^	1.366 (4)	C5—H5B	0.9700
C1—C2	1.380 (4)	C6—C7	1.505 (5)
C1—H1	0.9300	C6—H6A	0.9700
C2—O1	1.372 (3)	C6—H6B	0.9700
C2—C3	1.383 (4)	C7—C8	1.467 (5)
C3—C1^i^	1.366 (4)	C7—H7A	0.9700
C3—H3	0.9300	C7—H7B	0.9700
C4—O1	1.427 (3)	C8—C9	1.472 (6)
C4—C5	1.490 (4)	C8—H8A	0.9700
C4—H4A	0.9700	C8—H8B	0.9700
C4—H4B	0.9700	C9—H9A	0.9600
C5—C6	1.484 (4)	C9—H9B	0.9600
C5—H5A	0.9700	C9—H9C	0.9600
			
C3^i^—C1—C2	119.7 (3)	C7—C6—H6A	108.8
C3^i^—C1—H1	120.2	C5—C6—H6B	108.8
C2—C1—H1	120.2	C7—C6—H6B	108.8
O1—C2—C1	124.7 (3)	H6A—C6—H6B	107.7
O1—C2—C3	116.0 (2)	C8—C7—C6	115.0 (4)
C1—C2—C3	119.3 (3)	C8—C7—H7A	108.5
C1^i^—C3—C2	121.1 (2)	C6—C7—H7A	108.5
C1^i^—C3—H3	119.5	C8—C7—H7B	108.5
C2—C3—H3	119.5	C6—C7—H7B	108.5
O1—C4—C5	107.4 (3)	H7A—C7—H7B	107.5
O1—C4—H4A	110.2	C7—C8—C9	115.2 (5)
C5—C4—H4A	110.2	C7—C8—H8A	108.5
O1—C4—H4B	110.2	C9—C8—H8A	108.5
C5—C4—H4B	110.2	C7—C8—H8B	108.5
H4A—C4—H4B	108.5	C9—C8—H8B	108.5
C6—C5—C4	114.6 (3)	H8A—C8—H8B	107.5
C6—C5—H5A	108.6	C8—C9—H9A	109.5
C4—C5—H5A	108.6	C8—C9—H9B	109.5
C6—C5—H5B	108.6	H9A—C9—H9B	109.5
C4—C5—H5B	108.6	C8—C9—H9C	109.5
H5A—C5—H5B	107.6	H9A—C9—H9C	109.5
C5—C6—C7	113.9 (3)	H9B—C9—H9C	109.5
C5—C6—H6A	108.8	C2—O1—C4	118.7 (2)
			
C3^i^—C1—C2—O1	−178.8 (2)	C5—C6—C7—C8	177.1 (4)
C3^i^—C1—C2—C3	0.7 (4)	C6—C7—C8—C9	−179.4 (4)
O1—C2—C3—C1^i^	178.9 (2)	C1—C2—O1—C4	6.9 (4)
C1—C2—C3—C1^i^	−0.7 (4)	C3—C2—O1—C4	−172.7 (2)
O1—C4—C5—C6	175.7 (2)	C5—C4—O1—C2	170.4 (2)
C4—C5—C6—C7	−177.6 (3)		

Symmetry code: (i) −*x*, −*y*+1, −*z*+2.
